# A novel VOC breath tracer method to evaluate indoor respiratory exposures in the near- and far-fields; implications for the spread of respiratory viruses

**DOI:** 10.1038/s41370-022-00499-6

**Published:** 2022-11-23

**Authors:** Hooman Parhizkar, Mark Fretz, Aurélie Laguerre, Jason Stenson, Richard L. Corsi, Kevin G. Van Den Wymelenberg, Elliott T. Gall

**Affiliations:** 1 Institute for Health and the Built Environment, University of Oregon, Portland, OR 97209 USA; 2 Energy Studies in Buildings Laboratory, University of Oregon, Eugene, OR 97403 USA; 3 Biology and the Built Environment Center, University of Oregon, Eugene, OR 97403 USA; 4 Department of Mechanical and Materials Engineering, Portland State University, Portland, OR 97201 USA; 5 Department of Civil and Environmental Engineering, University of California, Davis, Davis, CA 95616 USA

**Keywords:** Bioaerosol, COVID-19, Healthy buildings, Risk, Infectious disease, PTR-ToF-MS

## Abstract

**Background:**

Several studies suggest that far-field transmission (>6 ft) explains a significant number of COVID-19 superspreading outbreaks.

**Objective:**

Therefore, quantifying the ratio of near- and far-field exposure to emissions from a source is key to better understanding human-to-human airborne infectious disease transmission and associated risks.

**Methods:**

In this study, we used an environmentally-controlled chamber to measure volatile organic compounds (VOCs) released from a healthy participant who consumed breath mints, which contained unique tracer compounds. Tracer measurements were made at 0.76 m (2.5 ft), 1.52 m (5 ft), 2.28 m (7.5 ft) from the participant, as well as in the exhaust plenum of the chamber.

**Results:**

We observed that 0.76 m (2.5 ft) trials had ~36–44% higher concentrations than other distances during the first 20 minutes of experiments, highlighting the importance of the near-field exposure relative to the far-field before virus-laden respiratory aerosol plumes are continuously mixed into the far-field. However, for the conditions studied, the concentrations of human-sourced tracers after 20 minutes and approaching the end of the 60-minute trials at 0.76 m, 1.52 m, and 2.28 m were only ~18%, ~11%, and ~7.5% higher than volume-averaged concentrations, respectively.

**Significance:**

This study suggests that for rooms with similar airflow parameters disease transmission risk is dominated by near-field exposures for shorter event durations (e.g., initial 20–25-minutes of event) whereas far-field exposures are critical throughout the entire event and are increasingly more important for longer event durations.

**Impact statement:**

- We offer a novel methodology for studying the fate and transport of airborne bioaerosols in indoor spaces using VOCs as unique proxies for bioaerosols. We provide evidence that real-time measurement of VOCs can be applied in settings with human subjects to estimate the concentration of bioaerosol at different distances from the emitter. We also improve upon the conventional assumption that a well-mixed room exhibits instantaneous and perfect mixing by addressing spatial distances and mixing over time. We quantitatively assessed the exposure levels to breath tracers at alternate distances and provided more insights into the changes on “near-field to far-field” ratios over time. This method can be used in future to estimate the benefits of alternate environmental conditions and occupant behaviors.

## Introduction

The spread of COVID-19 has caused extensive death and damage to the lives of millions of people worldwide. Severe acute respiratory syndrome coronavirus 2 (SARS-CoV-2), the causative agent of COVID-19, is transmitted from human to human via bioaerosol particles that are released during respiratory activities such as breathing, talking, singing, and coughing [1–3].

Epidemiological studies, public health research, and engineering risk assessment models of well-documented outbreaks indicate the important role that exposures beyond 2 m plays in COVID-19 disease transmission [3–10]. Therefore, quantifying the degree of exposure to bioaerosols according to distance from the source emitter is critical to characterize disease transmission risk more accurately, to determine the most effective environmental and human related risk reduction strategies such as ventilation, filtration, spatial distancing, and masking to reduce disease transmission.

A well-mixed air space is a conventional assumption that has been used in most studies of indoor air pollution and infectious disease transmission modeling [11]. For a well-mixed condition, indoor air contaminants, including virus laden aerosol particles, are assumed to be uniformly distributed by appropriate ventilation, interior mixing fans, buoyancy driven flows, and infiltration, immediately after being emitted from the source [12]. However, thermal stratification, low mixing flow rates from ventilation, and other environmental conditions can cause a non-uniform distribution of bioaerosols in indoor spaces [13, 14], where the probability of susceptible occupants inhaling virus-laden aerosol particles will rely, at least to some extent, on the distance from the source emitter. Moreover, the well-mixed assumption does not address sequences of mixing over time relative to emission rates and spatial parameters.

Few studies have considered the importance of spatial parameters such as room height into measurements of indoor pollutants [15–18]. A study of temporal and spatial scales suggests that chemical compounds as well as particles in the range of 1 µm −10 µm with persistent residence time exhibit spatial gradients that are significantly controlled by ventilation rates [19]. Additionally, controlled experiments with subjects diagnosed with COVID-19 were used to study the abundance of SARS-CoV-2 viral RNA copies in room aerosols. The authors found that the near-field was associated with a higher number of virus RNA copies, and statistically higher carbon dioxide (CO_2_), and particle counts of 0.3 µm – 2.5 µm than in the far-field [3]. Differences between near-field and far-field were also examined through the comparison of CO_2_ and particles with patients receiving high-flow nasal cannula therapy (HFNC), where the CO_2_ concentration was statistically higher at a distance 0.5 m (~1.6 ft) from the source emitter compared to background levels [20].

CO_2_ has been historically used as a tracer for estimating the concentration of human sourced indoor air contaminants as well as outdoor ventilation rate, and more recently discussed in the context of aerosol transmission of infectious disease [21, 22]. While CO_2_ can be a useful metric for estimating the rate of outdoor air intake, it cannot be applied as a uniquely identifiable bioaerosol tracer from a specific source emitter in a room with multiple people.

Real-time measurement of tracer compounds uniquely associated with an individual’s emitted breath offers the ability to directly study the transport, mixing, and exposure implications of exhaled breath constituents (e.g., pathogens) from only those individuals tagged as infectious. In this pilot study, we “tag” the individual’s respiratory emissions with high quantities of volatile tracers (emitted from breath mints) to serve as a proxy measurement of exposure of susceptible individuals to the “infected” individual’s respiratory emissions. The capability to measure tracers unique to only infectious individuals extends and complements exposure studies that have historically relied on CO_2_ to estimate rebreathed fractions of exhaled breath from all occupants in the space. Since both infectious and susceptible occupants emit CO_2_ and contribute to the rebreathed fraction, a large proportion of exhaled CO_2_ does not represent a potential risk of infection. Our approach allows measurement of a proxy of the contribution of respiratory emissions from infectious occupants. Our approach also offers the potential to study the dynamics of exposure risk by monitoring the “tagged” tracer concentrations in realistic indoor spaces occupied by any number of individuals, and any subset of infectious individuals, through high time resolution measurements of VOCs throughout an indoor space. These measurements enable study of transport and mixing effects on exposure to exhaled breath tracers in complex fluid flows empirically. This is in contrast to commonly used models, e.g., the Wells-Riley [23] model, that rely on an assumption of perfect mixing in the space.

The goals of the present study are to (a) determine the effectiveness of tracer compounds sourced from breath mints consumed by a participant and (b) to better characterize the impact of distance from the emission source on distribution of exhaled bioaerosol in an indoor environment.

## Methodology

### Background

A previous study has shown that chewing peppermint flavored gum is associated with release of unique volatile organic compounds (VOCs) such as menthone and menthol, with source strength dependent on oral activity and chewing frequency [24]. Real-time measurements of VOCs can provide useful information for studying pollutant dynamics of indoor environments [25]. We used proton transfer reaction - time of flight - mass spectrometry (PTR-ToF-MS) to measure VOCs associated with breath mints across a mass range of 17–490 amu with 1 second time resolution.

### PTR-ToF-MS

The principles of the PTR-ToF-MS measurements have been described previously [26–28].This approach allows for a real-time measurement of VOCs with a proton affinity greater than that of H_2_O. In theory, ionization is soft, allowing for little fragmentation, and compound identification can be made by observation of the [M + H] + ion (i.e., molecular mass + the mass of the transferred proton). Our study used breath mints instead of chewing gum to monitor compounds that are predominantly associated with breath emissions from the mint: menthone, monoterpenes, and menthol. These compounds were shown to be substantially elevated in exhaled breath of the participant when consuming a breath mint. We putatively identify the signal m/z 155.150 as protonated menthone and m/z 139.137 as dehydrated menthol [29, 30]. Monoterpenes are a class of compounds that share the chemical formula C_10_H_16_; our PTR-ToF-MS is unable to distinguish isomers. We report monoterpenes as the sum of signals m/z = 137.144 and the known fragment m/z = 81.070; our study was conducted under similar ionization conditions to prior studies that show monoterpenes fragmentation at these signals without interferences [31, 32]. We sum the concentration from those two ions and report as monoterpenes.

### Participant recruitment

Human subject protocols were reviewed and approved by the University of Oregon Institutional Review Board (IRB) (Protocol #20210509). One human subject participated in this study. The participant was a 25–30 year old male with a height of 1.89 m (6.1 ft) and a sitting height of 1.2 m (3.6 ft). The participant was instructed to:

- not use cologne or body sprays during the day preceding and during the study period;
- wear clothes that were not recently washed with detergents;
- follow a consistent diet during the course of three data collection days;
- maintain a constant breath mint consuming rate during all trials.

### Climate chamber

**Fig. 1:**
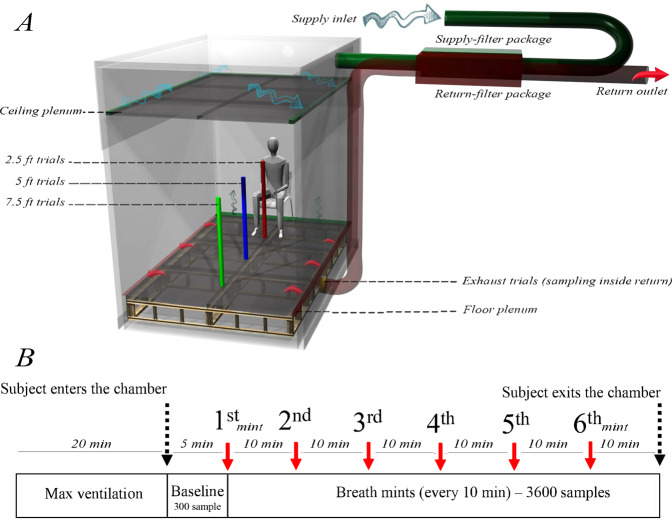
Digital model of the Experimental setup indicating. **A** climate chamber, airflow distribution, as well as sampling location for each unique trial (modeled in Rhinoceros software), (**B**) experimental procedure and the number of breath mints consumed by the participant for each trial.

Experiments were conducted at the Energy Studies in Buildings Laboratory, Portland, OR, USA, using a custom environmentally-controlled climate chamber with an interior volume of 27 m^3^ (Fig. 1A). Filtered air was supplied from the lab’s environment through a ceiling plenum and exhausted to the lab’s environment through a floor plenum. Both supply and exhaust air were filtered by activated carbon beds. Air was exchanged at ~3 air changes per hour (ACH) during test periods and flushed at >20 ACH for a minimum of 20 minutes between trial periods. Three air changes per hour was selected to ensure that steady-state conditions would be approached by the end of each 60-minute trial. We observed the concentration of breath tracers during the experiment to confirm the removal of previous residuals before the beginning of each trial.

Ambient indoor air was supplied through a MERV-13 pre-filter and high-flow activated carbon filters (Air Box 4 Stealth; AirBox Filters, Laval, Quebec, CA) and exhausted through an identical filtration system (shown as supply filter package and return filter package in Fig. 1A). Air change rate was monitored throughout the experiment using in-duct thermal anemometer probes and multi-function ventilation meters (#964 and #9565-P, respectively; TSI Incorporated, Shoreview, MN, USA).

### Experimental procedure

Each trial began with adjustment of the climate chamber’s ventilation rate to the maximum value (20 ACH) for a minimum duration of 20 minutes without the presence of the participant in order to evacuate residual VOCs from prior trials (Fig. 1B). We monitored the concentration of menthone to assure it reached a negligible steady-state background concentration. Next, the participant was instructed to enter the chamber, sit in a chair, and breathe normally for five minutes without consuming any breath mints. These 5-minute periods provided a baseline reference for each trial and were included in the study protocol to identify certain compounds that are exclusively associated with natural human breath and not breath mint flavoring, and to additionally provide a baseline to observe any exhaled compounds that may have remained in the participant’s mouth from previous trials. After 5 minutes, the participant was visually informed to begin consuming one breath mint every 10 minutes (Fig. 1B), resulting in 6 breath mints consumed during each 1-hour trial (minutes 0, 10, 20, 30, 40, and 50). Through some qualitative experiments, we identified that it takes ~10 minutes until 1 mint is fully dissolved in the participant’s mouth while sitting and breathing normally. Therefore, the participant was instructed to consume breath mints as consistently as possible (without chewing) during 10-minute intervals controlled by a timer. All breath mints were carried into the chamber by the participant in an air sealed plastic bag. To keep emissions relatively constant, the participant was instructed to remain silent and minimize body movement during the entire course of study. The participant also maintained a resting activity level between trials to avoid emission irregularities while inside the chamber during the trials.

A summary of all trials conducted in this study is presented in Table 1. We used a single sampling line attached to a portable tripod at participant’s breathing zone level (1.2 m) and moved the probe after each 1-hour trial to designated spots on the floor, measuring 0.76 m (2.5 ft), 1.52 m (5 ft) and 2.28 m (7.5 ft) from the participant’s mouth (Table 1, Trials A-C). Additionally, we placed another sampling line of equal length inside the floor plenum exhaust duct (called exhaust trials) to measure exhaust air as the volume-averaged concentration (Table 1, Trial D). Each trial (Table 1, trials A-D) lasted for 1-hour during which PTR-ToF-MS continuously measured VOCs at 1-second resolution, resulting in 3600 samples for each trial. For each location, we measured the concentration of VOCs in duplicate trials with random order over the course of a 3-day sampling period. The concentrations of each compound for duplicate trials at each sampling location were averaged to produce a single data set for each of the distance trials (0.762 m, 1.524 m, 2.28 m, and exhaust trials).

**Table 1:** Summary of all experiment trials.

Trials	Sampling probe distance from the participant’s mouth	Number of replicates	Sampling frequency (Hz)	Sampling duration (minutes)	Number of samples
A	0.76 m (2.5 ft)	2	1	60	3600
B	1.52 m (5 ft)	2	1	60	3600
C	2.28 m (7.5 ft)	2	1	60	3600
D	Exhaust	2	1	60	3600
E	Breath mint in a 250 ml glass container	1	1	20	1200
F	Breath mint exhaled into a 250 ml glass container	1	1	20	1200

In addition to trials A-D (Table 1), we conducted two other experiments to confirm the presence of unique tracer compounds associated with the exhaled breath of the participant consuming breath mints (Table 1, Trials E&F). In order to confirm which compounds were natively sourced from the breath mint we conducted trial E. In Trial E we placed one single breath mint in the headspace of a 250 mL glass container for ~1 minute and monitored the concentration of VOCs over a 20-minute period. For similar reasons, for trial F the participant was instructed to consume one breath mint while breathing normally into the same 250 mL glass container for ~1 minute. For both trial E and F (Table 1), we monitored the concentration of VOCs over a 20-minute period using PTR-ToF-MS sampled at a flowrate of ~100 cc/min during the experiments. Three minutes of background (BCK) measurements were taken prior to the start of the experiment.

### Statistical analysis

Analyses were performed using the statistical programming environment R. The Taylor expansion [33] procedure was applied using the propagate package [34] to calculate the expanded uncertainties associated with VOC measurements. The ratio of samples collected at 0.76 (2.5 ft), 1.52 (5 ft), and 2.28 m (7.5 ft) were normalized by the volume-averaged concentration (VAC) resulting in a series of magnifiers for each distance expressed in percentage values. The effect size associated with each magnifier was assessed using the Cohen’s d test [35, 36].

## Results

Menthone, menthol, monoterpenes, isoprene, and acetone were considered for analysis. We conducted paired *t* test analyses between the first and last minute of baseline periods during which the participant did not consume breath mints in the chamber (n = 60). Table 2 presents the results of paired t-tests between the first and last minute of the baseline periods for each distance. The concentration of menthone, menthol, and monoterpenes did not change (*p* > 0.05) during baseline periods when the participant did not consume breath mints, while the concentration of isoprene and acetone changed during the baseline periods. This indicates that acetone and isoprene were detected in the participant’s natural breath which is consistent with previous findings [37]. However, changes in isoprene and acetone were inconsistent with breath sources only, suggesting other indoor sources such as the participant’s skin and climate chamber interior materials may have contributed to the variability. Therefore, we summed the concentrations of menthone, menthol, and monoterpenes as a unique breath tracer for the comparison of different distances in this study.

**Table 2:** Comparison of the first and last minute of baseline period for five major compounds (paired *t* test) for trials A-C (Table 1).

	Sampling distance from human source emitter (*n* = 60)		
Compounds	0.76 m (2.5 ft)	1.52 m (5 ft)	2.28 m (7.5 ft)
Menthone	0.0005(p = 0.92)	−0.005 (p = 0.32)	0.01 (p = 0.1)
Menthol	0.0086(p = 0.46)	0.02 (p = 0.35)	−0.019 (p = 0.07)
Monoterpenes	0.0015(p = 0.78)	0.0015 (p = 0.76)	−0.0015 (p = 0.79)
Isoprene	0.07(p < 0.005)	−0.055 (p < 0.001)	−0.049 (p < 0.001)
Acetone	0.59(p < 0.001)	−0.59 (p < 0.001)	−0.34 (p < 0.001)

**Fig. 2:**
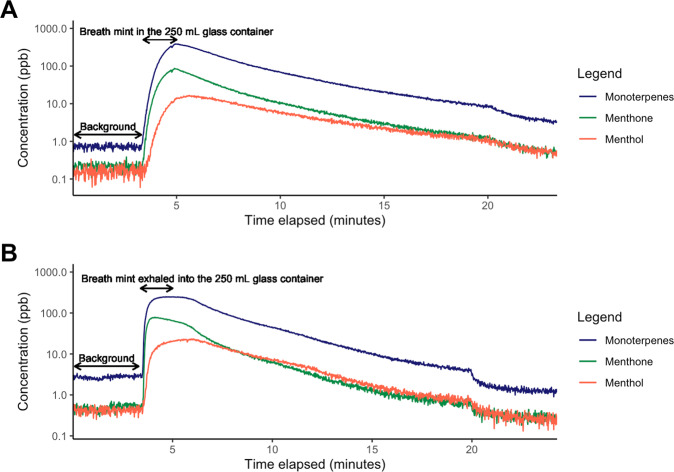
The concentration of breath mint in the headspace of a 250 mL glass container. **A** Concentration of breath tracer compounds (menthol, menthone, and monoterpenes) in the headspace of a 250 mL glass chamber as a function the time when a breath mint is placed inside, (**B**) Concentration of the three target compounds when the participant exhaled their breath once into the 250 mL chamber while consuming the breath mint.

In addition to the analysis of baseline periods presented in Table 2, the presence of menthone, menthol, and monoterpenes in the exhaled breath of the participant while consuming breath mints was additionally confirmed through trials E & F (Table 1), occurring in a 250 mL glass flow-through glass chamber. Figure 2 indicates that the concentrations of menthone, menthol, and monoterpenes substantially increase when the breath mint was placed in the headspace of a 250 ml container (Fig. 2A), and when the participant breathed naturally into the 250 ml container while consuming the mint (Fig. 2B). We subsequently refer to the summation of the above-mentioned signals (menthone, menthol, and summed monoterpenes) as “breath tracer compounds.” We estimated the emission rate of breath tracer compounds for the first ~30 seconds of trials E & F (Table 1) as VOCs accumulated in the 250 mL flow-through glass chamber at ~4.8 air change per minutes (Fig. 2B). We estimate the emission rate of breath tracer compounds to be ~130 µg/h (monoterpenes = 90 µg/h, menthone = 37 µg/h, and menthol = 5 µg/h). These values are an order of magnitude greater than the total endogenous emission of these species determined by Wang et al. [38], implying measured breath tracer compounds here are the dominant emissions from the subject.

**Fig. 3:**
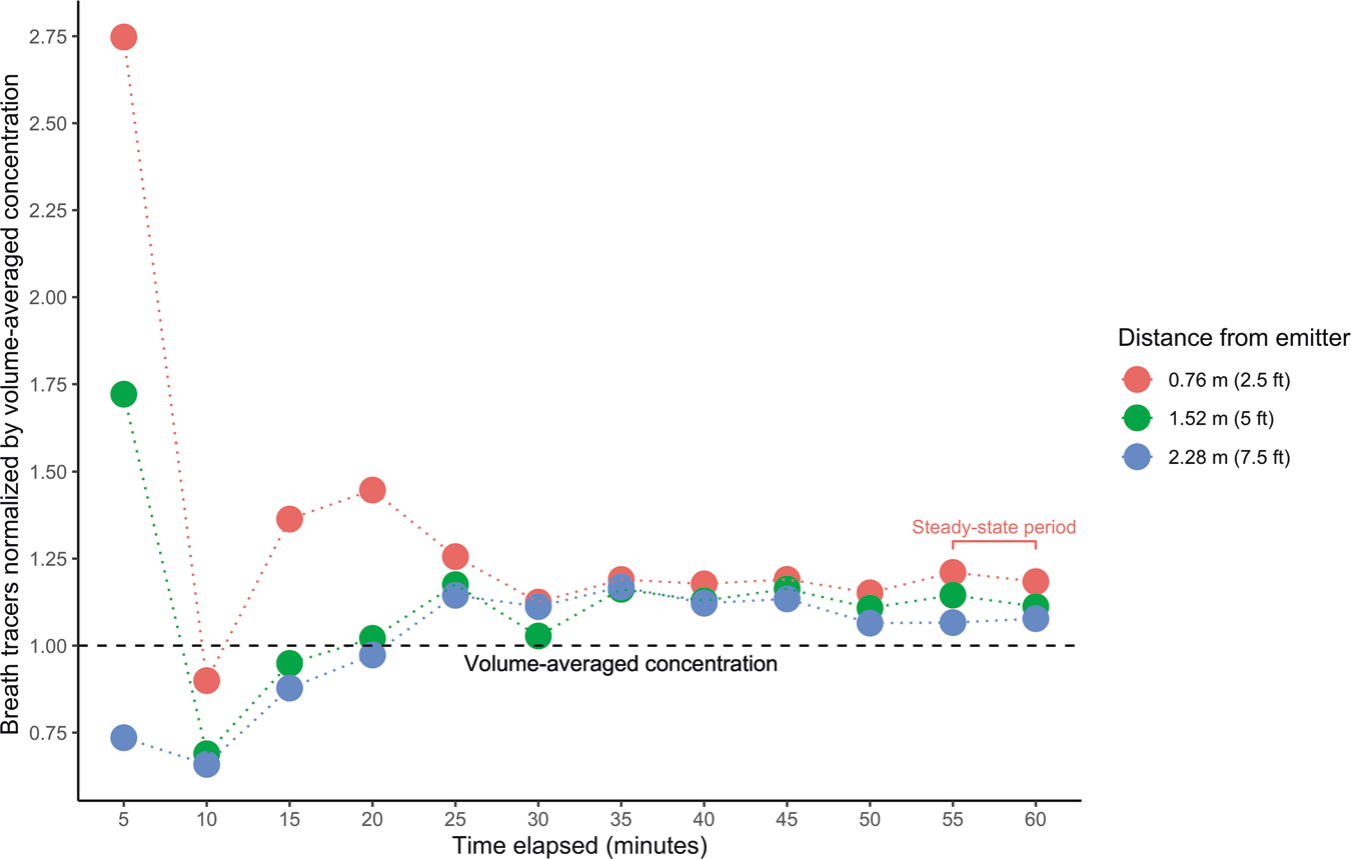
Comparison of 0.762 m (2.5 ft), 1.524 m (5 ft), 2.28 m (7.5 ft) trials normalized by volume-averaged concentration (VAC) whereby values below 1.0 indicate concentrations proportionally lower than the VAC at that time point, and values higher than 1.0 indicate concentrations proportionally higher than the VAC at that time point. Note that VAC changes over time.

Furthermore, Supplementary Fig. 1 shows the concentration of major VOCs during ventilation and baseline periods, indicating low but not necessarily zero concentrations for trials A-D (Table 1). We hypothesize that baseline concentrations are associated with residual VOCs that were adsorbed by climate chamber or ventilation filter surfaces and slowly re-emitted into the chamber air (Supplementary Table 1). To make a more consistent starting point for all trials, we subtracted the average concentration of each compound detected during baseline periods (minutes −5 to 0) from the 1-hour trials (minutes 0–60). A comparison of summed breath tracer compounds normalized by the volume-averaged concentration is shown for each distance in Fig. 3.

Table 3 reports on the magnifiers, expanded uncertainties associated with each value, as well as Cohen’s d effect size statistics for each distance. Supplementary Figure 2 demonstrates the uncertainties associated with the values presented in Fig. 3. Supplementary Figure 3 shows the time series concentration of breath tracer compounds over time for all 4 locations.

**Table 3:** Magnifier, effect size (Cohen’s d), and expanded uncertainty (Taylor Expansion) values 0.762 m (2.5 ft), 1.524 m (5 ft), 2.28 m (7.5 ft) normalized by volume-averaged concentrations in 5 minutes time step.

	0.762 m (2.5 ft)			1.52 m (5 ft)			2.28 m (7.5 ft)		
Intervals	Magnifier	Effect size*	±EU^2^	Magnifier	Effect size*	±EU^2^	Magnifier	Effect size*	±EU^2^
Min_0_60	1.21	0.32 (s)	0.34	1.07	0.14 (N)	0.32	1.05	0.1 (N)	0.3
Min_0_5	2.74	0.57(M)	4.68	1.72	−0.08(N)	3.49	0.73	−0.58(M)	1.88
Min_5_10	0.89	−0.26(S)	0.5	0.68	−0.95(L)	0.51	0.65	−1.12(L)	0.43
Min_10_15	1.36	1.96(L)	0.42	0.94	−0.42(S)	0.34	0.87	−0.93(L)	0.30
Min_15_20	1.44	3.65(L)	0.37	1.02	0.09(N)	0.31	0.97	−0.28(S)	0.27
Min_20_25	1.25	2.00(L)	0.3	1.17	1.32(L)	0.29	1.14	1.07(L)	0.26
Min_25_30	1.12	1.39(L)	0.23	1.02	0.23(S)	0.22	1.11	1.08(L)	0.21
Min_30_35	1.19	2.13(L)	0.25	1.16	1.78(L)	0.25	1.16	2.12(L)	0.22
Min_35_40	1.17	2.00(L)	0.23	1.12	1.35(L)	0.21	1.12	1.41(L)	0.21
Min_40_45	1.19	2.54(L)	0.2	1.16	2.27(L)	0.21	1.13	1.88(L)	0.19
Min_45_50	1.15	2.39(L)	0.18	1.1	1.71(L)	0.18	1.06	1.01(L)	0.17
Min_50_55	1.21	2.76(L)	0.25	1.14	2.01(L)	0.21	1.06	0.92(L)	0.2
Min_55_60^1^	1.18	2.39(L)	0.25	1.11	1.49(L)	0.21	1.07	1.03(L)	0.17

*N = negligible effect size, S = small effect size, M = medium effect size, L = large effect size.

^1^ = Steady-state period.

^2^ = Expanded uncertainty.

## Discussion

We used PTR-ToF-MS to trace the concentration of breath tracer compounds associated with a consumed breath mint as a proxy for bioaerosol emissions from a healthy participant during each 60-minute trial. For each trial, we summed the concentrations of menthone, sum of monoterpenes, and menthol at the resolution of each sample as a unique breath tracer since they were detected only when the participant consumed breath mints. The summed tracer concentrations detected at 0.76 m (2.5 ft), 1.524 m (5 ft), and 2.28 m (7.5 ft) from the participant were normalized by VAC, which indicates the magnifier of each location compared with an approximate well-mixed condition. As shown in Fig. 3, the concentration of VOCs at 0.76 m (2.5 ft) and 1.524 m (5 ft) remain above VAC during the first 5 minutes of the study, while the concentration at 2.28 m (7.5 ft) begins to approach the VAC after minute ~10 and exceed the VAC by minute ~20 (Fig. 3). We observed a steep increase in the concentration of breath tracer compounds at 0.76 m (2.5 ft), 1.52 m (5 ft), and 2.28 m (7.5 ft) during the first five minutes, which indicates that signals at short distances exceeded those measured at the exhaust plenum, likely due to a concentrated exhaled plume that had not mixed extensively throughout the chamber. At minute 5, the concentration of breath tracer compounds also began to rise in the exhaust plenum (VAC), resulting in decreases in the ratios (multipliers) of indoor sampling locations normalized by the VAC (Fig. 3). Shortly after minute 10, the concentration of breath tracer compounds during 0.762 m (2.5 ft) trials exceed the VAC, resulting in a higher concentration at 0.762 m (2.5 ft) during minutes 5–20 compared to all other locations and having a 36–44% higher concentration than VAC. This finding suggests that the risk of exposure to virus-laden aerosol particles during the first 20 minutes in close vicinity of a source within a space similar to our chamber is relatively higher for close contact distances (less than 0.91 m) when compared with other distances. Meanwhile, the concentration of breath tracer compounds at 1.52 m (5 ft) and 2.28 m (7.5 ft) also rise above VAC during minutes 20–25, with the greatest magnifier having a value 17% higher than the VAC at 1.52 m (5 ft). After 25 minutes, tracer concentrations at all distances maintained a relatively consistent relationship relative to the VAC. However, sampling locations further from the occupant progressively approached background concentrations. The magnifiers during the final 5-minutes of experiments were ~18% (±25%, *Cohen*’*s d estimate* = *Large*), ~11% (±21%, *Cohen’ s d estimate* = *Large*), and 7.5%(±18%, *Cohen’ s d estimate* = *Large*) above VAC at 0.76 m (2.5 ft), 1.52 m (5 ft), and 2.28 m (7.5 ft), respectively. The expanded uncertainties associated with values reported in Table 3 are in agreement with previous studies that measured VOCs using PTR-ToF-MS [39]. Despite the fact that Cohen’s d statistics show large effect size values when the concentration of breath tracer compounds at 0.76 m (2.5 ft), 1.52 m (5 ft), and 2.28 m (7.5 ft) were compared to VAC [40], the uncertainty associated with our measurements suggest that reported magnifiers presented in Table 3 should be studied further with more replicates to improve the accuracy of, and confidence in, the near-field to far-field multipliers. Meanwhile, these findings highlight the importance of both near-field and far-field exposure events and emphasize the importance of exposure duration in consideration of near-field and far-field mitigation priorities. In this study, and given these room characteristics and airflow rates, the airborne disease transmission risk associated with the initial period of source emissions appears to be dominated by near-field exposures, whereas far-field exposure risks are increasingly important as the event precedes.

### The implications of magnifiers in a real-world case study

The purpose of this section is to compare the results of near -field and far-field magnifiers for the present study and two recent relevant studies [3, 20]. In one study [20] the team measured near-field and far-field CO_2_ concentrations to estimate magnifiers in patient rooms within a healthcare environment having 8–11 ACH. The authors reported background (far-field) CO_2_ concentrations of 580 ppm (mean across 7 patients) and reported near-field mean CO_2_ concentrations of 605 ppm, thus only 25 ppm higher than background, which equates to a near-field magnifier of 4.3% [20]. We note that the uncertainty in CO_2_ measurements for most systems reported in the literature is ±50 ppm.

A second study focused on bioaerosols emitted from individuals diagnosed with COVID-19 within a space having similar environmental conditions as the chamber used for the present study (Supplemental Table 2) [3]. Near-field (1.2 m, 4 ft) and far-field (3.5 m, 11 ft) designations were used to report the concentration of SARS-CoV-2 viral RNA in room aerosols, CO_2_, and particles in the range of 0.3–25 µm. We translate their data into near-/far-field ratios to provide a comparison with the near-field magnifiers reported in Fig. 3 of the present study. The near-/far-field ratios from the previous study (Supplemental Table 3) ranged from ~8–12% for CO_2_ and particles (1–2.5um), which correspond reasonably well with the near-field magnifiers of the present study (Fig. 3), where the concentration of targeted VOCs in the near-field (0.762 m) was ~10% higher than far-field (2.28 m) during steady-state periods.

In summary, the concentration of CO_2_ and the particles of 1 µm – 2.5 µm in the controlled study on participants who were diagnosed with COVID-19 [3] were ~8% and ~12% higher in the near field, 1.2 m (4 ft), compared to the far field, 3.5 m (11 ft), respectively [3]. The concentration of targeted VOCs in the near-field (0.762 m) was ~10% higher than the far-field (2.28 m) during steady-state periods, thus- providing greater confidence for the concept of breath tracers as a proxy for virus laden bioaerosols.

## Discussion and limitations

Our study provides evidence that a novel breath tracer approach has utility in bioaerosol experiments when paired with a PTR-ToF-MS. This study also provides a series of magnifiers that could be used to estimate the concentration of bioaerosols at 0.76 m (2.5 ft), 1.52 m (5 ft), and 2.28 m (7.5 ft) from a human emitter in a reasonably well-mixed indoor space with conditions similar to those used in chamber experiments described in this paper. While these magnifiers are specific to this environmental chamber and ventilation pattern, the methodology can support future microbial risk assessment models that superimpose near-field exposures and inhalation dose on far-field exposures estimated using a well-mixed assumption.

Our findings suggest that the concentration of bioaerosols in the far-field is relatively close to the volume-averaged concentration at steady-state, while close range distances are associated with relatively higher exposure levels during the first 20 minutes of an emission exposure event.

Our study was a pilot project with several limitations. Our data were limited to duplicate trials and a constant ventilation rate of ~3 ACH in a chamber with a specific volume, ventilation system, and environmental conditions. Future research should include variations in related chamber conditions as well as multiple human participants in controlled settings to study the impact of occupant related factors such as thermal plumes and human motions on the concentration and dispersion of tagged breath tracers around people’s breathing and alternate positions. Ventilation strategies other than overhead (used in this experiment) should be considered with different ACH in future efforts. We seek to study several other distances & positions from the source emitter (vertical and horizonal distribution) to improve the accuracy of magnifiers with the intention of developing a comprehensive heterogeneous air space model for indoor air quality research.

## Supplementary information

Reporting Checklist

Supplementary information

## Data Availability

All data and code supporting this study and required to create the analyses are provided in Github, available at https://github.com/BioBE/Breath_Tracer.

## Institutional Review

All protocols relating to human subjects involved in the study were reviewed and approved by the University of Oregon Institutional Review Board (IRB) (Protocol #20210509).
