# Supplementary material for: A novel VOC breath tracer method to evaluate indoor respiratory exposures in the near- and far-fields; implications for the spread of respiratory viruses
Source: J Expo Sci Environ Epidemiol. 2022 Nov 23;33(3):339–46. doi: 10.1038/s41370-022-00499-6 (PMC9686220; doi:10.1038/s41370-022-00499-6)
Supplement: Supplementary file 1 — Reporting Checklist [file 41370_2022_499_MOESM1_ESM.pdf]

SPRINGER NATURE

Corresponding Author name: Kevin Van Den Wymelenberg

Manuscript Number: JESEE-22-3957.R1

Reporting Checklist

This checklist is used to ensure the quality, transparency, and reproducibility of published results. We require authors attest that these components have been considered and addressed.

**Exposure Assessment Guiding Principle**	**Yes/No/Not Applicable**
Has the method to estimate exposure been described clearly?	Yes
Has the exposure assessment method been validated/evaluated as a proxy for exposure and is its validity or agreement with other methods described?	Yes
Is the time period over which the exposure assessment method is considered to be a proxy for exposure appropriate for the research question?	Yes
If exposure is modeled or measured, were all critical potential routes and sources of exposure considered?	Not Applicable
If exposure is modeled, how does it vary over space and time and are necessary historical data incorporated?	Not Applicable
If biomarkers are used as indicators of exposure, could the biomarker measurement have been affected by the outcome (i.e., reverse causality)?	Not Applicable
Are the strengths and weaknesses of the exposure approach detailed and discussed?	Yes
