# Supplementary material for: A novel VOC breath tracer method to evaluate indoor respiratory exposures in the near- and far-fields; implications for the spread of respiratory viruses
Source: J Expo Sci Environ Epidemiol. 2022 Nov 23;33(3):339–46. doi: 10.1038/s41370-022-00499-6 (PMC9686220; doi:10.1038/s41370-022-00499-6)
Supplement: Supplementary file 2 — Supplementary information [file 41370_2022_499_MOESM2_ESM.pdf]

A novel VOC breath tracer method to evaluate indoor respiratory exposures in the near- and far-fields; Implications for the spread of respiratory viruses

Hooman Parhizkar[1,2], Mark Fretz[1,2,3], Aurélie Laguerre[4], Jason Stenson[1,2], Richard L. Corsi[5], Kevin G. Van Den Wymelenberg[1,2,3*], Elliott T. Gall[4]

1 - Institute for Health and the Built Environment, University of Oregon, Portland, OR, United States, 97209

2 - Energy Studies in Buildings Laboratory, University of Oregon, Eugene, OR, United States, 97403

3 - Biology and the Built Environment Center, University of Oregon, Eugene, OR, United States, 97403

4 - Department of Mechanical and Materials Engineering, Portland State University, Portland, OR, United States, 97201

5 - Department of Civil and Environmental Engineering , University of California, Davis, Davis, CA, United States, 95616

***Corresponding Author:** Kevin G. Van Den Wymelenberg, kevinvdw@uoregon.edu, (541) 346-5647, Biology and the Built Environment Center, University of Oregon, Eugene, OR, United States, 97403, Energy Studies in Buildings Laboratory, University of Oregon, Eugene, OR, United States, 97403, Institute for Health and the Built Environment, University of Oregon, Portland, OR, United States, 97209

Supplemental Figure 1 Evaluating the impact of distance on bioaerosol exposure in a typical indoor environment

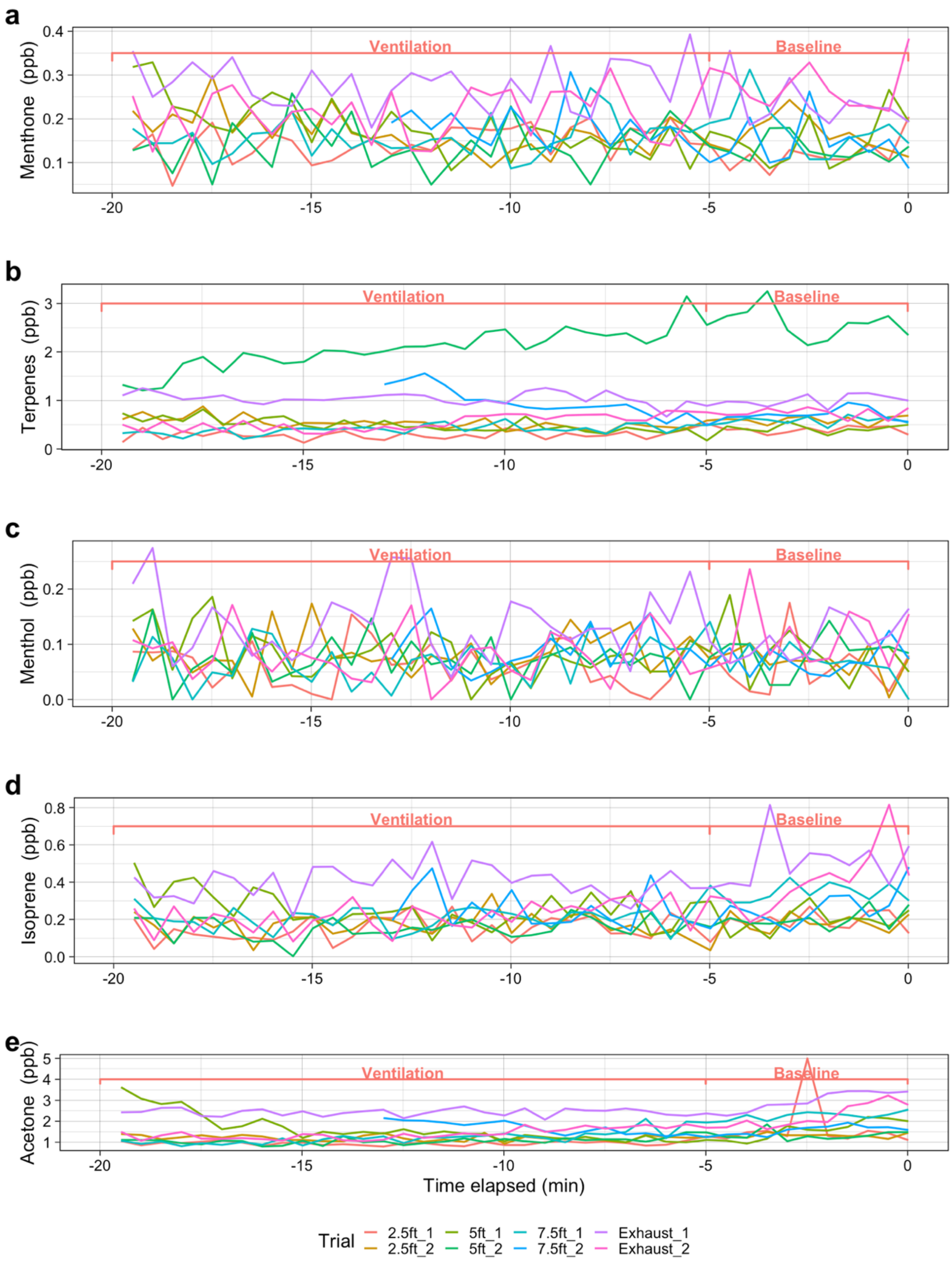

Supplemental Table 1 The ratio of background concentrations compared with peak values for three major compounds

Ratio of background/peak	Menthone	Menthol	Monoterpenes
0.76 m (2.5 ft) trial 1	0.14	0.21	0.14
0.76 m (2.5 ft) trial 2	0.12	0.19	0.14
1.52 m (5 ft) trial 1	0.11	0.19	0.10
1.52 m (5 ft) trial 2	0.12	0.23	0.61
2.28 m (7.5 ft) trial 1	0.13	0.23	0.17
2.28 m (7.5 ft) trial 2	0.17	0.25	0.20
Exhaust plenum trial 1	0.27	0.44	0.34
Exhaust plenum trial 2	0.26	0.38	0.26

Supplemental Figure 2. Comparison of the concentrations of breath tracers for each distance to volume integrated background with expanded uncertainties.

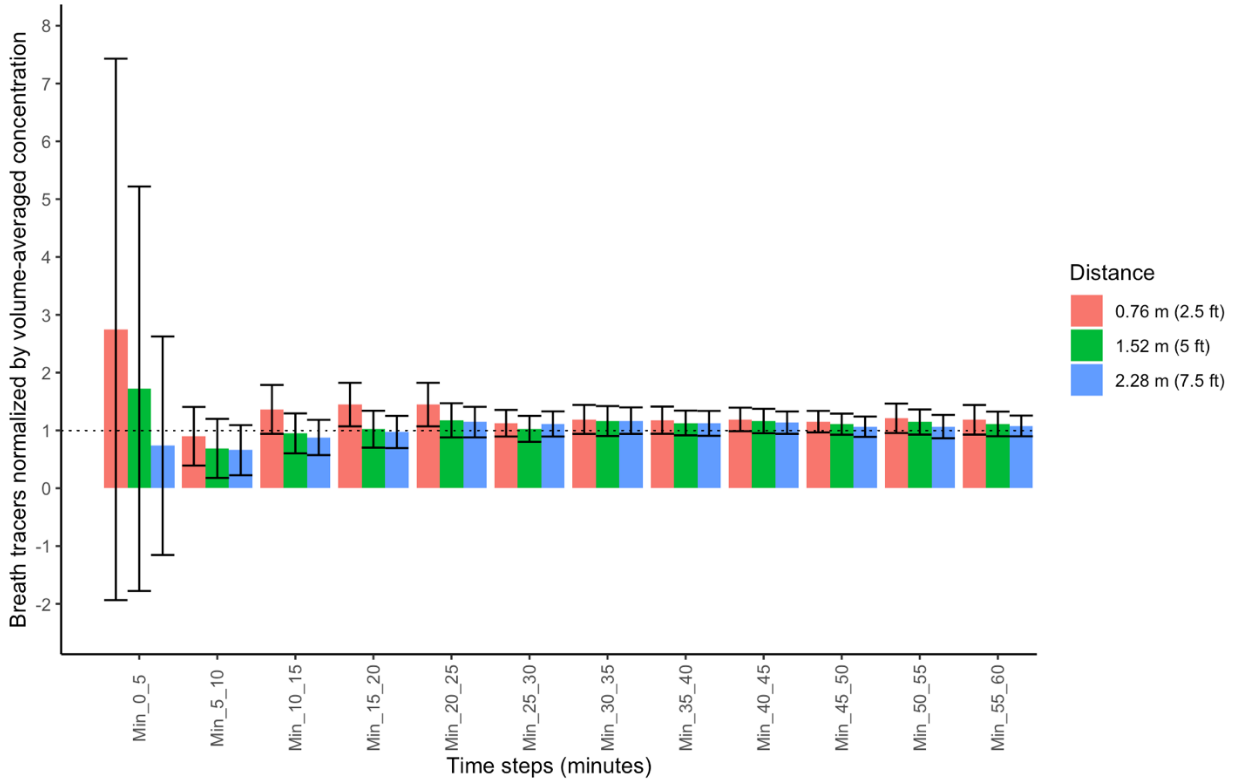

Supplemental Figure 3. The time series concentration of breath tracer compound over time in 5 seconds resolution for each location

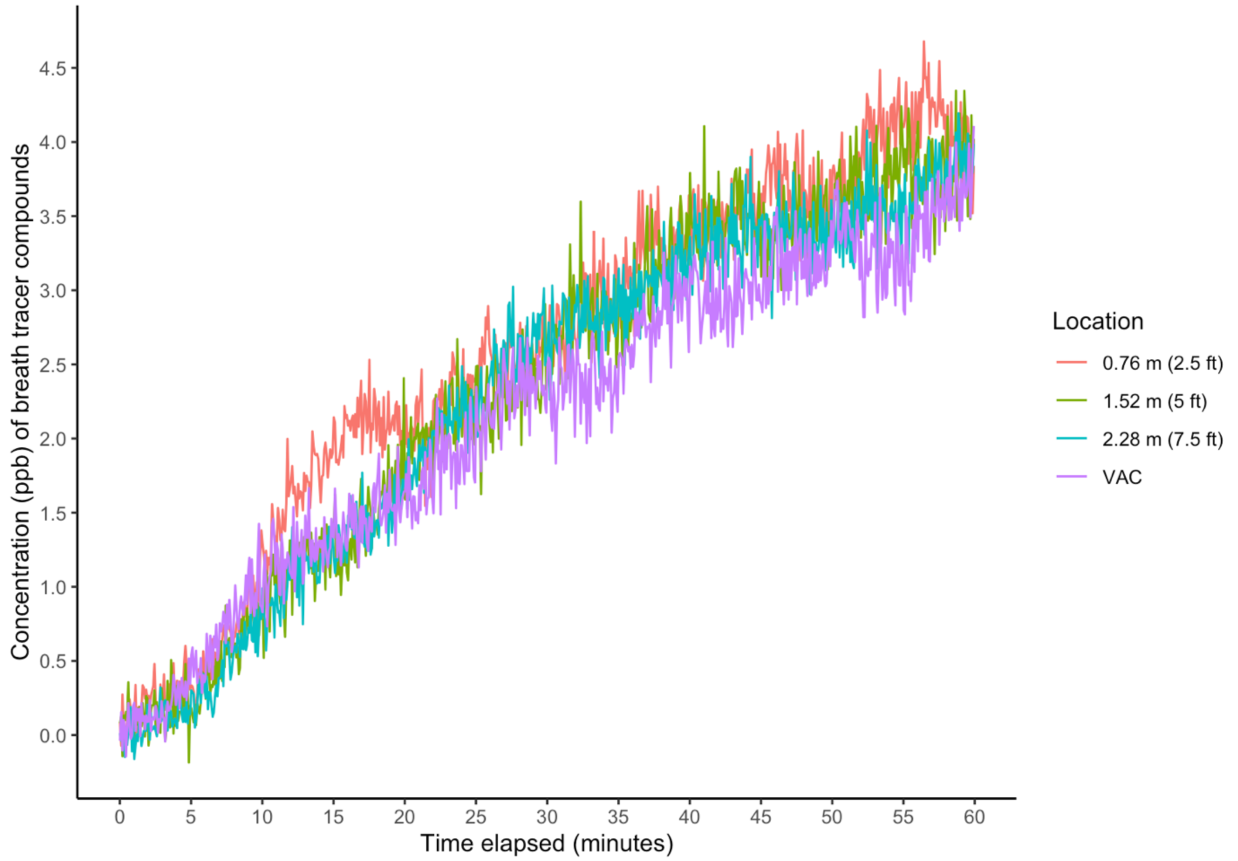

Supplemental Table 2 Comparison of spatial parameters between the present and the controlled study with participants diagnosed with COVID-19.

Key variables	Breath tracer (present study)	Parhizkar, et al (2022)
**Volume (m3)**	27	28.04
**Air exchange rate (1/h)**	~ 3 ACH	Nearly 0
**Duration (minutes)**	60	60
**Near- field distance (ft)**	0.76 (2.5 ft)	1.2 (4 ft)
**Far- field distance m (m)**	2.28 (7.5 ft)	3.5 (11 ft)
**Near/far fields ratio**	3	2.75
**Number of participants in the room for each trial**	1	1

Supplemental Table 3 Comparison of near- field and far-field in a recent controlled study on participants that were diagnosed with COVID-19.

**Variable**	*Near-field*	*Far-field*	**Near-field / Far-field**
CO_2 (ppm)	937.72	862.41	**1.08**
Particles, 0.3 µm - 1µm	16454.36	15959.25	**1.03**
Particles, 1 µm - 2.5 µm	559.51	493.71	**1.12**
Particles, 2.5 µm - 3µm	37.61	31.71	**1.17**
Particles, 3 µm - 5 µm	50.86	45.12	**1.11**
Particles, 5 µm - 10 µm	26.47	27.24	N/A
Particles, 10 µm - 25 µm	8.85	8.96	N/A

Reference

1. Parhizkar H, Dietz L, Olsen-Martinez A, et al. Quantifying environmental mitigation of aerosol viral load in a controlled chamber with participants diagnosed with COVID-19. *Clin Infect Dis*. January 2022. January 6, 2022. https://academic.oup.com/cid/advance-article/doi/10.1093/cid/ciac006/6498295. Accessed January 17, 2022.

2. Gall ET, Laguerre A, Noelck M, Van Meurs A, Austin JP, Foster BA. Near-field airborne particle concentrations in young children undergoing high-flow nasal cannula therapy: a pilot study. *J Hosp Infect*. 2021;113:14–21.
